# Evaluation of non-instrumented nucleic acid amplification by loop-mediated isothermal amplification (NINA-LAMP) for the diagnosis of malaria in Northwest Ethiopia

**DOI:** 10.1186/s12936-015-0559-9

**Published:** 2015-01-28

**Authors:** Meslo Sema, Abebe Alemu, Abebe Genetu Bayih, Sisay Getie, Gebeyaw Getnet, Dylan Guelig, Robert Burton, Paul LaBarre, Dylan R Pillai

**Affiliations:** Department of Medical Laboratory Sciences, College of Medicine and Health Sciences, Wollo University, Dessie, Ethiopia; School of Medicine, College of Health Sciences and Medicine, Wolaita Sodo University, Wolaita, Ethiopia; Department of Pathology and Laboratory Medicine, University of Calgary, Calgary, Canada; Department of Medical Parasitology, School of Biomedical and Laboratory Sciences, College of Medicine and Health Sciences, University of Gondar, Gondar, Ethiopia; PATH, Seattle, USA

## Abstract

**Background:**

Malaria is a major public health problem in sub-Saharan African countries including Ethiopia. Early and accurate diagnosis followed by prompt and effective treatment is among the various tools available for prevention, control and elimination of malaria. This study aimed to evaluate the performance of non-instrumented nucleic acid amplification loop-mediated isothermal amplification (NINA-LAMP) compared to standard thick and thin film microscopy and nested PCR as gold standard for the sensitive diagnosis of malaria in Northwest Ethiopia.

**Methods:**

A cross-sectional study was conducted in North Gondar, Ethiopia from March to July 2014. Eighty-two blood samples were collected from malaria suspected patients visiting Kola Diba Health Centre and analysed for *Plasmodium* parasites by microscopy, NINA-LAMP and nested PCR. The NINA-LAMP method was performed using the Loopamp™ Malaria Pan/Pf detection kits for detecting DNA of the genus *Plasmodium* and more specifically *Plasmodium falciparum* using an electricity-free heater. Diagnostic accuracy outcome measures (analytical sensitivity, specificity, predictive values, and Kappa scores) of NINA-LAMP and microscopy were compared to nested PCR.

**Results:**

A total of 82 samples were tested in the primary analysis. Using nested PCR as reference, the sensitivity and specificity of the primary NINA-LAMP assay were 96.8% (95% confidence interval (CI), 83.2% - 99.5%) and 84.3% (95% CI, 71.4% - 92.9%), respectively for detection of *Plasmodium* genus, and 100% (95% CI, 75.1% - 100%) and 81.2% (95% CI, 69.9% - 89.6%), respectively for detection of *P. falciparum* parasite. Microscopy demonstrated sensitivity and specificity of 93.6% (95% CI, 78.5% - 99.0%) and 98.0% (95% CI, 89.5% - 99.7%), respectively for the detection of *Plasmodium* parasites. *Post-hoc* repeat NINA-LAMP analysis showed improvement in diagnostic accuracy, which was comparable to nested PCR performance and superior to microscopy for detection at both the *Plasmodium* genus level and *P. falciparum* parasites.

**Conclusion:**

NINA-LAMP is highly sensitive for the diagnosis of malaria and detection of *Plasmodium* parasite infection at both the genus and species level when compared to nested PCR. NINA-LAMP is more sensitive than microscopy for the detection of *P. falciparum* and differentiation from non-falciparum species and may be a critical diagnostic modality in efforts to eradicate malaria from areas of low endemicity.

## Background

Malaria is an infectious disease caused by protozoan parasites of the genus *Plasmodium* that continues to exact a large human toll in endemic areas [1]. Although the incidence and malaria specific mortality rate is declining worldwide due to concerted global malaria control efforts, malaria remains a major public health issue in sub-Saharan African countries with occasional epidemics leading to significant mortality. Children less than five years of age and pregnant mothers bear the greatest burden of illness [2-4].

Malaria contributes to 12% of outpatient consultations and 10% hospital admissions in Ethiopia [5,6]. To reduce this impact, the Ethiopian government is implementing a five-year National Strategic Plan for Malaria Control and Prevention, starting 2011. Achieving zero malaria transmission in malarious areas and malaria elimination in low transmission areas of the country are the two major goals of the strategic plan. To achieve these goals, the strategy calls for early and accurate diagnosis followed by prompt treatment and case management of patients with malaria [7,8].

Clinical diagnosis and parasitological confirmation by microscopy using Giemsa-stained blood films (‘Giemsa microscopy’) or rapid diagnostic test (RDT) are the malaria diagnostic approaches currently employed throughout Ethiopia. Giemsa microscopy is considered the gold standard and RDTs are alternatively used for the diagnosis of malaria in all health facilities or through rural health extension and outreach. RDTs are relatively easier to perform and used for screening of malaria in remote areas where electricity and other resources are limited [9,10]. However, microscopy and RDTs cannot reliably detect lower-density parasitaemia (<100 parasites/μL) [11]. Moreover, microscopy requires experience and intensive training on the part of the microscopist and needs careful preparation and application of reagents to ensure quality control and assurance [12-14].

A recent study in Ethiopia showed that a high rate of sub-microscopic *Plasmodium* parasite infection was detectable by polymerase chain reaction (PCR) [15]. Another study also showed that, compared to nested PCR, microscopy resulted in a high degree of misidentification and misclassification of *Plasmodium* parasites in Ethiopia [16]. Similarly, the RDT methods reveal inconsistency of performance (sensitivity, 20% to 99%) and stability problems in rural health facilities where storage temperatures may exceed 30°C [14].

Recently, nucleic acid amplification tests (NAATs) are being considered as a point of care test (POCT) for diagnosis of malaria. These methods can detect the presence of parasite in low-level infections which otherwise would be missed by microscopy or RDT [17]. NAATs are used for the detection of submicroscopic infections and to increase the power of surveys at low transmission settings [18]. Nested PCR is commonly used for malaria epidemiological surveys with a detection limit of ~0.2 parasites/μL blood [19]. However, the method is prone to contamination and reagents must be stored in cold conditions to preserve function. The technique is also sophisticated, requires training, capital investment and expensive reagents. Therefore, PCR assays are less feasible to be used as a POCT for malaria diagnosis in developing countries where malaria is endemic. Because of its high sensitivity and specificity, PCR assays have recognized value in research settings and can serve as reference method in the evaluation of other diagnostic methods [17,20].

Loop-mediated isothermal amplification (LAMP) can amplify DNA/RNA with high specificity, efficiency and rapidity under isothermal conditions. The method employs DNA polymerase and four or six primers recognizing distinct gene sequences targeting mitochondrial DNA of the parasite. The method can detect parasitaemia as few as 5 parasites/μl of blood, below the detection limit of microscopy or RDT [21,22].

Numerous attempts have been made to develop simplified molecular diagnostics for malaria appropriate for low-resource or resource compromised settings [23,24]. PATH has recently developed a variety of non-instrumented nucleic acid amplification (NINA) heater configurations to facilitate pathogen detection via isothermal nucleic acid amplification assays, such as LAMP. The low-cost, electricity-free, reusable NINA platform heater enables pathogen detection in low-resource settings where there is no access to electricity and/or instrumentation [25-28]. LAMP executed in a NINA heater is rapid and simple and can be accomplished by minimally-trained health workers. Results can be read simply by observing fluorescence or turbidity visually in the reaction tube with no additional processing [21,29,30].

In Ethiopia, availability of more rapid, easy, sensitive and specific method of diagnosis is crucial to the success of the National Strategic plan to eradicate malaria. The diagnostic performance of LAMP has not yet been evaluated as a laboratory diagnosis tool in Ethiopia. This study sought to examine the diagnostic performance of NINA-LAMP compared to microscopy and nested PCR for the diagnosis of malaria at Kola Diba Health Centre, northwest Ethiopia.

## Methods

### Study design and study area

A cross-sectional diagnostic evaluation study was conducted at Kola Diba Health Centre, Dembia District of North Gondar administrative zone, northwest Ethiopia. The district has an altitude range between 1,750 and 2,100 m above sea level and lies close to Lake Tana. Malaria is the most prevalent seasonal disease in the area and accounts for the second most common reported disease in the health centre. *Plasmodium vivax*, *Plasmodium falciparum* and *Plasmodium ovale* are all reported in the area [31,32]. Malaria clinical diagnosis is based on Giemsa microscopy in the health centre.

### Study subjects and inclusion criteria

Study participants were recruited consecutively (convenient sampling) from malaria suspected febrile outpatients based on self-reported history of fever within the previous 24 hours and referred to the laboratory for malaria testing using microscopy. Male and female febrile patients of any age were enrolled in the study. Study participants were not involved in the decision to be referred to the laboratory or in any decision regarding clinical management. Patients who had received anti-malarial drugs during the past four weeks and critically ill patients were excluded from participation. Since the prevalence of malaria during data collection (March to April) was very low, a total of 200 study participants were recruited to increase the number of positive cases for assuring the reliability of diagnostic test evaluation. All microscopy positives (30) and 52 negatives from a total 170 negatives were included in the study. The 52 negatives were selected by identifying one in every three (approximately) microscopy negative patients for molecular testing by NINA-LAMP and nested PCR.

### Blood collection and microscopic diagnosis of malaria

Capillary blood was taken from 200 study participants, and both thick and thin blood film was prepared on a slide for microscopic detection of *Plasmodium* parasites. After air-drying, the thin blood films were fixed in methanol. Thin and thick blood films were stained with 10% Giemsa solution for 10 min and examined by experienced laboratory personnel using manual for laboratory diagnosis of malaria in Ethiopia (2012). The presence of *Plasmodium* infection was ruled out if no parasites were observed after examining at least 100 microscopic fields with 100X objective. Parasitaemia was estimated by counting the number of parasites per 200 white blood cells in a thick blood film and then calculated as parasite count per microlitre by assuming a total specimen white blood cell count of 8,000/μl [33].

Giemsa microscopy was performed by two experienced laboratory technicians and verified by a study-blinded third expert to resolve any discordance between the two readers. The reported parasite density is the average of the two laboratory personnel’s parasite count. If more than 10% discrepancy was observed between the two readers parasite count, the third expert blindly counted parasite load and recorded as correct result.

### Sample collection for molecular analysis

Approximately four millilitres of venous blood was drawn from 82 microscopically confirmed study participants (30 positives and 52 negatives participants). Soon after collection, two separate drops of blood were placed on Whatman filter paper 903 (GE Healthcare) and air dried individually to avoid any chance of contamination. The remaining blood was dispensed into 5 ml tubes containing 0.08 ml of 10% ethylenediaminetetraacetic acid (EDTA) solution and stored at 2-8°C in Kola Diba health centre up to four days. The patient code and date of collection were recorded on filter paper and EDTA tube. Samples collected at the health centre were transported once a week to the University of Gondar laboratory on ice. Upon arrival, venous blood samples were stored in −80°C freezer for subsequent LAMP analysis. The dried filter papers were individually inserted into small zip locked plastic bags and packed within a larger plastic bag for transportation to University of Calgary, Canada for nested PCR analysis.

### Malaria LAMP assay using NINA

The LAMP assay used in this study utilize primers for amplification of parasite mitochondrial DNA. Loopamp™ malaria Pan/Pf detection kits (Eiken Chemicals, Tokyo, Japan) consisting of plastic reaction tubes containing thermostable vacuum-dried reagents used to amplify *Plasmodium/ P. falciparum* DNA. The parasite DNA was extracted by a boil and spin method as follows. Sixty μL of EDTA blood was added to 60 μL of extraction solution (400 mM NaCl, 40 mM Tris pH 6.5, and 0.4% sodium dodecyl sulfate) in an Eppendorf tube, heated for 5 minutes at 95°C with a water bath, and centrifuged at 10,000 × g for 3 minute; 30 μL of the clear supernatant was pipetted into a dilution tube containing 345 μl sterile water. Then, 30 μl of diluted DNA sample was dispensed into the reaction tube and mixed well with reagent for use in the NINA-LAMP assay.

**Figure 1:**
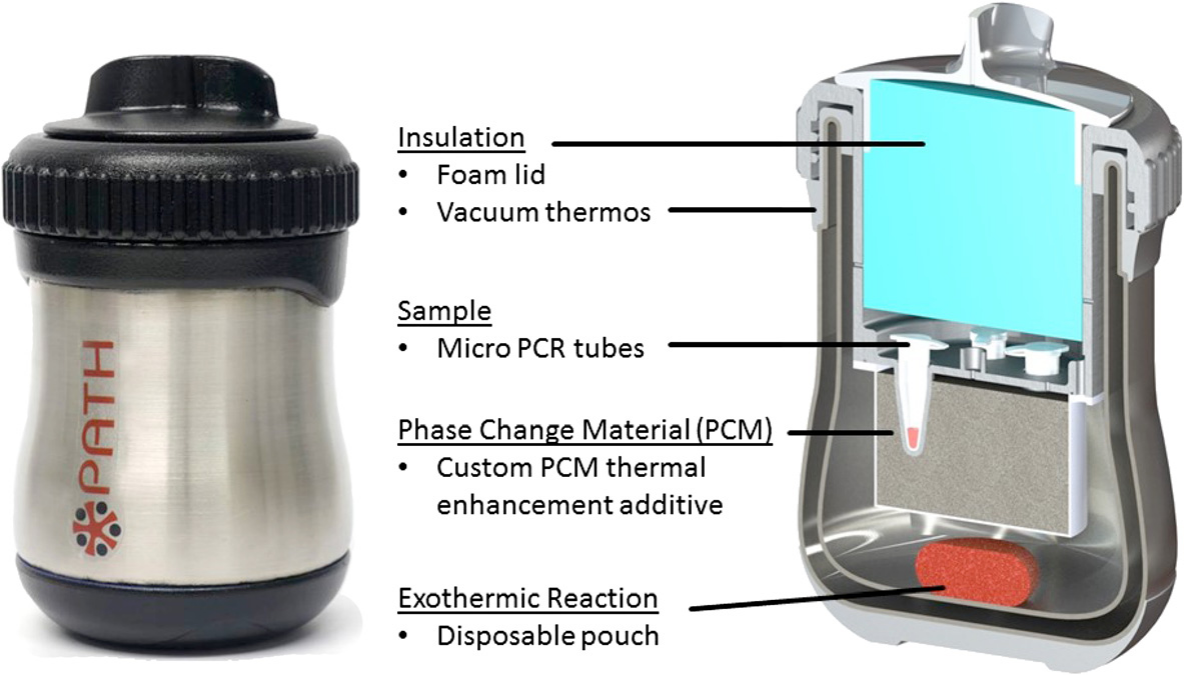
**Photograph and cross sectional view of the NINA H.V6 prototype heater device.** The reusable-housing platform is designed to heat micro PCR tubes using a commercial thermos with manufactured inserts. Five sample wells are surrounded by the phase change material (PCM) chamber and the exothermic reaction takes place below the PCM at the bottom of the stainless-steel insulated thermos. The PCM is used to buffer the exothermic reaction and provide a constant temperature to the sample wells. Disposable magnesium iron alloy (MgFe) and saline cartridges can be simply dropped into the housing to activate the device.

The NINA H.V6 prototype heater was used to produce isothermal conditions suitable for LAMP procedure, after which the tubes were removed and analysed. The NINA device operation was performed following the prototype manual provided by PATH (Seattle, USA). An exothermic chemical reaction coupled with a phase-change material (PCM) provides temperature control to the NINA device (Figure 1). To activate the heater, 5 mL 0.9% saline (Medline Industries Inc, USA) is combined with 0.9 g MgFe fuel pack, provided by PATH inside of the thermos cup. Post-activation, the device requires approximately 15 minutes for reaching isothermal conditions of amplification after initiation of exothermic reaction and maintains 64 ± 1°C for 60 minutes. DaqPRO 5300 data recorder containing instrumented PCR tubes was used to measure and record temperature profile of the device. Reaction tubes containing extracted DNA sample and reagent were inserted into HV6 thermos to be amplified after checking the internal temperature (63–65°C). After 40 minutes of amplification, the reaction tubes were removed from device. The LAMP reaction produces insoluble magnesium orthophosphate as a by-product of DNA amplification, which is detected visually as turbidity immediately post-reaction.

The NINA-LAMP assay was conducted by one laboratory technician following adequate training in the test procedure. Sample processing and LAMP result interpretation were performed following standard protocols determined previously by the Foundation for Innovative New Diagnostics (FIND) [34]. To avoid cross-contamination, three separate rooms were use to perform DNA extraction, mixing of reagents and sample, and amplification using standard one-way workflow. Upon completion of the reaction, LAMP amplification test results were read and interpreted by observing turbidity using positive and negative control results as comparator standards. Two additional study-blinded laboratory technicians read the turbidity observed at the end of the LAMP reaction and the consensus was regarded as the correct interpretation of LAMP result. Laboratory personnel performing the LAMP assay were blinded to any result of Giemsa microscopy and nested PCR during the course of the study.

### Nested PCR- based detection of *Plasmodium* parasites

Genomic DNA was extracted from dried blood on Whatman filter paper 903 (GE Healthcare). Two six millimetre diameter blood containing filter paper confetti were cut from each sample using a paper puncher and transferred into a microtube. To prevent sample cross-contamination, the puncher was cleaned with 70% ethanol, sterilized using the flame of a Bunsen burner and two confettis were cut from a new filter paper before taking the next sample. Genomic DNA was extracted using Quick-gDNA™ Blood MiniPrep kit (Zymo research corp., USA). The procedure on the company’s instruction manual was followed with slight modification. Briefly, the blood spot confetti were treated with 200 μl of genomic lysis buffer for 30 minutes with quick vortex every five minutes. The DNA in the buffer was then transferred into Zymo-Spin™ IIC Column in a collection tube, washed with wash buffer and eluted with 40 μl of sterile distilled water.

Nested PCR was performed following previously published procedures with slight modification [35,36]. The small subunit ribosomal RNA gene was amplified using genus-specific primers (rPLU5 and rPLU6). Then, the PCR product was diluted 1:5 in sterile distilled water. The second PCR was done from the diluted PCR product in four separate tubes using species-specific primers; rFAL1/rFAL2, rVIV1/rVIV2, rOVA1/OVA2, rMAL1/rMAL2, for *P. falciparum*, *P. vivax*, *P. ovale*, and *Plasmodium malariae*, respectively. The PCR mixture contained 1X standard Taq buffer, 125 nmol dNTPs, 2.5 μM MgCl_2_, 250nMol of each of the forward and reverse primers, 0.4U Taq DNA polymerase, sterile distilled water, and template DNA in a total volume of 20 μl. All the reagents were purchased from New England BioLabs® Inc. (Whitby, ON). The first and second PCR reactions were carried out using 5 μl of the genomic DNA and 2 μl of the diluted first PCR product, respectively. The PCR program for the first PCR involved one cycle of 95°C for 3 min, 58°C for 2 min, 68°C for 2 min followed by 24 cycles of denaturation at 94°C for 1 min, annealing at 58°C for 2 min, and extension at 68°C for 2 min and a final cycle of 58°C for 2 min and 68°C for 5 min. The second PCR was run with the same program but 30 cycles. The final PCR product was then run on a 2% agarose gel and visualized using a Gel Doc™ (Bio-Rad, Mississauga, ON).

### Data analysis

After assuring data completeness, data were analysed by SPSS version 20 and MedCalc easy to use online statistical software version 13.3 used for diagnostic test evaluation. Sensitivity, specificity, and positive and negative predictive values of NINA- LAMP and Giemsa microscopy were determined using nested PCR as the gold standard for diagnosis of malaria. The concordance response rate (percentage of responses with both positive and both negative results) and *Kappa* value (k) was determined to measure degree of agreements between two diagnostic test results. Secondary analysis (repeat testing from original samples) was performed at University of Calgary for samples which the original nested PCR and malaria NINA-LAMP results disagreed.

### Ethical consideration

The study protocol was reviewed and approved by Research and Ethical Review Committee of School of Biomedical and Laboratory Sciences, College of Medicine and Health Sciences, University of Gondar (SBLS; reference No 525/06). Permission to conduct the study was also obtained from Dembia district health bureau. All study participants signed written informed consent before enrollment. Patients found to be positive for malaria parasite were treated according to the current treatment guideline for malaria in the country.

## Results

### Parasite positivity by Giemsa microscopy and NINA-LAMP and nested PCR

Giemsa microscopy for 200 febrile malaria suspected patients resulted in 15.0% (30/200) confirmed positive for *Plasmodium* parasites with a median parasite density of 9,800 parasites/μL (parasitaemia range = 420–186,800 parasites/μL). Of those positives, 43.3% (13/30) and 56.7% (17/30) had *P. falciparum* and *P. vivax* infection, respectively. Mixed infection with *Plasmodium* species was not identified by microscopy. From the total of 200 study participants, samples of 82 patients (30 positives and 52 negatives) were analysed by NINA-LAMP and nested PCR methods. During primary analysis, NINA-LAMP method identified 38 positives from total 82 samples performed in Gondar using Loopamp™ malaria Pan Detection kits (46.3%). From those positives by NINA- LAMP, 68.4% (26/38) tested positive for *P. falciparum* using Loopamp™ malaria Pf detection kits. The nested PCR analysis performed using four *Plasmodium* species primers detected 31 positives for *Plasmodium* parasites from 82 patient samples analysed. Of those positives, 41.9% (13/31), 35.5% (11/31) and 22.6% (7/31) were due to *P. falciparum, P.vivax and P. ovale,* respectively. All *P. ovale* positive samples diagnosed by nested PCR were misclassified as *P vivax* by microscopy. Table 1 presents the parasite positivity by Giemsa microscopy, NINA- LAMP and nested PCR for the diagnosis of *Plasmodium* parasites.

**Table 1:** **NINA- LAMP result compared with Giemsa-stained microscopy and nested PCR for the detection of** ***Plasmodium*** **parasite in 82 patient samples at Kola Diba health centre, Northwest Ethiopia, 2014**

**Giemsa microscopy (** ***n*** **)**	**Primary NINA- LAMP result (n)**	**Nested PCR (n)**
Positives (30),	Positives (38),	Positives (31),
*P. falciparum *(13)	*P. falciparum *(26)	*P. falciparum (13)*
*P.vivax *(17)	Non- *P. falciparum *(12)	*P. vivax (11), P. ovale *(7)
Negatives (52)	Negatives (44)	Negatives (51)

n = number of blood samples examined; NINA = Non-instrumented nucleic acid amplification; LAMP = Loop-mediated isothermal amplification; nested PCR = nested polymerase chain reaction.

### Discordant analysis of microscopy, NINA-LAMP and nested PCR for diagnosis of *Plasmodium* parasites

During primary NINA-LAMP analysis in Gondar, false positives were observed in detecting both genus *Plasmodium* and *P. falciparum.* Discordant results were observed from ten patient samples using the three diagnostic tests for detecting *Plasmodium* parasites in Gondar. The NINA-LAMP method identified eight patient samples as positive for *Plasmodium* parasites, which were classified as negative by microscopy. Nested PCR detected one positive and seven negative from those discordant results by NINA-LAMP and Giemsa microscopy. Additionally, one sample was identified as positive by nested PCR from previously negative samples by microscopy and NINA–LAMP. Nested PCR also detected one negative result from other positive sample by Giemsa microscopy and NINA-LAMP. The NINA-LAMP method detected 13 additional positive samples for *P. falciparum* using nested PCR as reference method (Table 2). In *post hoc* analysis, nested PCR and NINA-LAMP discordant samples were retested in a controlled laboratory setting in the University of Calgary, Canada. Specifically, NINA-LAMP was repeated for fourteen samples to detect *Plasmodium* genus and/or *P. falciparum*. All samples with primary positive results in Gondar were subsequently identified as NINA-LAMP negative for *Plasmodium* genus and *P. falciparum*. After NINA-LAMP repeat testing, only results of two samples were discordant with results of nested PCR for detecting genus *Plasmodium* (Table 2).

**Table 2:** **Discordant analysis of microscopy, NINA-LAMP and nested PCR for detection of** ***Plasmodium*** **parasite at Kola Diba Health Centre, northwest Ethiopia, 2014**

	**Microscopy and nested PCR (−), NINA LAMP (+)**	**Microscopy and NINA-LAMP (+), nested PCR (−)**	**Microscopy and NINA-LAMP (−), Nested PCR (+)**	**Nested PCR and NINA-LAMP (+), Microscopy (−)**	**Negative result by three methods**
Primary analysis in Gondar for pan (n)	7	1	1	1	43
Primary analysis in Gondar for Pf (n)	13	0	0	1	56
Repeat testing in Calgary for pan (n)	0	1	1	1	50
Repeat testing in Calgary for Pf (n)	0	0	0	1	69

(−) means negative result, (+) means positive result, n = number of patient samples.

### Diagnostic accuracy of NINA-LAMP and Giemsa microscopy as compared to nested PCR for diagnosis of malaria

During primary analysis in Gondar, Ethiopia, the LAMP assay had a sensitivity and specificity of 96.8% and 84.3% respectively compared with the nested PCR method. Using Bayesian analysis, the positive and negative predictive values of NINA-LAMP for detecting *Plasmodium* parasites were 52.1% and 99.3%, respectively. The NINA-LAMP result had also substantial agreement (κ = 0.776) with results of nested PCR for detection of *Plasmodium* parasites. For the diagnosis of *P. falciparum*, the sensitivity and specificity of NINA-LAMP was 100% and 81.2%, respectively. The corresponding positive and negative predictive values were 27.0% and 100%, respectively. In this study, NINA-LAMP assay and nested PCR showed moderate agreement (κ = 0.577) for identification of *P. falciparum* parasite.

In a controlled laboratory setting in Calgary, Canada, the NINA-LAMP method showed improvements in diagnostic accuracy using nested PCR as reference method. The sensitivity, specificity, PPV and NPV of NINA-LAMP for detecting *Plasmodium* parasites were 96.8%, 98.0%, 89.7% and 99.4%, respectively. The NINA-LAMP result had almost perfect agreement (κ =0.948) with results of nested PCR for detection of *Plasmodium* parasites. The secondary NINA-LAMP test result was also 100% concordant (sensitivity, specificity, PPV and NPV of 100%) with nested PCR for detection of *P. falciparum.*

The sensitivity, specificity, PPV, and NPV of microscopy were 93.6%, 98.0%, 89.4% and 98.8% respectively compared with nested PCR for the diagnosis of *Plasmodium* parasites. The microscopy data agreed well (96.3%) with the nested PCR results. For the detection of *P. falciparum*, microscopy also demonstrated sensitivity, specificity, PPV and NPV of 92.3%, 100%, 100% and 99.5%, respectively. Table 3 presents sensitivity, specificity, negative predictive value, positive predictive value and Kappa value for NINA-LAMP assay and microscopy as compared to nested PCR for detection of genus *Plasmodium* and *P. falciparum* parasite.

**Table 3:** **Diagnostic accuracy of NINA-LAMP and microscopy compared with the gold standard nested PCR for diagnosis of** ***Plasmodium*** **parasite and** ***P. falciparum*** **at Kola Diba Health Centre, Northwest Ethiopia, 2014**

**Method**		**Senstivity(%) (95% CI)**	**Specificity(%) (95% CI)**	**PPV(%) (95% CI)**	**NPV(%) (95% CI)**	**%agreement (Kapp = κ** **)**
NINA -LAMP	Pan (Gondar)	96.8 (83.2-99.5)	84.3 (71.4-92.9)	52.1 (33.9-71.3)	99.3 (96.0-99.9)	89.0 (0.776)
	Pf (Gondar)	100 (75.1-100)	81.2 (69.9-89.6)	27.0 (14.8-40.0)	100 (97.6-100)	84.1 (0.577)
	Pan (Calgary)	96.8 (83.2-99.5)	98.0 (89.5-99.7)	89.7 (58.3-98.2)	99.4 (96.8-99.9)	97.6 (0.948)
	Pf (Calgary)	100 (75.1-100)	100 (94.7-100)	100 (49.8-100)	100 (98.2-100)	100 (1.000)
Microscopy	Pan	93.6 (78.5-99.0)	98.0 (89.5- 99.7)	89.4 (56.9-98.2)	98.8 (95.9-99.8)	96.3 (0.922)
	Pf	92.3 (63.9-98.7)	100 (94.7-100 )	100 (45.8-100)	99.5 (97.4-99.9)	98.8 (0.953)

Percentage of agreement between tests: (Number of positives by both tests + Number of negatives by both tests)/*N,* against what might be expected by chance; Kappa value(κ): 1.00 = Perfect agreement; > 0.80, almost perfect agreement; 0.61-0.8, substantial agreement; 0.41-0.6, moderate agreement; 0.21-0.4, fair agreement; NPV = negative predictive value; PPV = positive predictive value; Pan = *Plasmodium genus*; *Pf = Plasmodium falciparum*; 95% CI = 95% confidence interval.

## Discussion

Early and accurate diagnosis of malaria in health service facilities is required for prompt treatment and case management of malaria [9,10]. Routine diagnostic methods such as microscopy and RDT cannot detect low-density *Plasmodium* infections and have a number of practical limitations [11-14]. NAAT methods like LAMP have been developed and evaluated for diagnosis of malaria in different parts of the world. LAMP utility is enhanced by NINA as no major capital equipment or electricity is required to perform the reaction [26-28]. Performance evaluation of LAMP was conducted in different settings using nested PCR and/or microscopy as reference methods. Previous findings suggest that this molecular diagnostic kit would be used as point of care for detection of malaria [17,37].

The NINA- LAMP assay used pan–*Plasmodium* genus specific primers and *P. falciparum* specific primers. The pan–*Plasmodium* genus specific primers were used to confirm the presence of malaria by detecting mitochondrial DNA of *Plasmodium* genus in samples of symptomatic patients. This NINA-LAMP evaluation showed good diagnostic accuracy for the diagnosis of malaria as compared to nested PCR, which satisfies the WHO recommendation that requires diagnostic kits sensitivity of greater than 95%. The LAMP performed in the NINA heater achieved diagnostic accuracy comparable to other LAMP evaluation studies conducted using nested PCR as the reference method. For example, using *Plasmodium* genus specific primers, LAMP showed sensitivity and specificity of 97.0%, 99.2%, respectively from samples extracted by PURE method in a UK parasitology reference laboratory for returning travellers [38]. The LAMP assay performed at remote clinic in Uganda using samples extracted by boil and spins method showed almost comparable sensitivity (93.9%) and lower specificity (76.3%) than the present study [39]. Furthermore, in Republic of São Tomé and Príncipe, LAMP demonstrated 100% sensitivity and 98% specificity for diagnosis of *Plasmodium* parasites before treatment using nested PCR as reference method [40]. Thus, the present study was in line with the diagnostic accuracy of LAMP reported in the previous studies.

For rapid case management and treatment of patients with malaria, confirming the presence of potentially fatal falciparum malaria is necessary. Loopamp™ Malaria Pf detection kits containing *P falciparum* primers were used to confirm the presence of this species. For the diagnosis of *P. falciparum*, the present NINA-LAMP method results were similar with diagnostic sensitivity of LAMP reported in Northern Thailand (sensitivity = 100%) and the UK reference laboratory (sensitivity = 98.4%) [38,41]. This study also showed far higher diagnostic sensitivity of LAMP than sensitivity reported in Bangladeshi in-patients with fever (sensitivity =76.1%) [42].

The diagnostic accuracy of LAMP in a secondary laboratory analysis was higher than the primary field analysis. The possible reason may arise from sample contamination during primary analysis. Primary analysis was performed on site in Gondar with limited laboratory materials and resources while secondary analysis was performed at University of Calgary with direct access to substantial laboratory resources. Due to limited resources, non-filter tips were used to transfer samples and reagents in Gondar, leading to suspected aspirate contamination of the pipettes. The boil and spin method of DNA extraction was performed in an open system which contributes to possible risks of contamination during sample processing in Gondar. As a result, false amplification in the negative control tube was observed in some LAMP tests during primary analysis. Simple remedial actions were taken in the form of repeated sterilization of tools and regents, and proper cleaning of work areas until false amplification in the negative control was eliminated. This suggests that temporary laboratory contamination occurred and may have resulted in the observed discordance between primary and secondary LAMP testing. Furthermore, no contamination issues arose from filter paper samples evaluated by LAMP in Calgary. Filter papers were split off and sent to Calgary prior to any possibility of end product contamination in Gondar.

A recent study in northwest Ethiopia showed that nested PCR is more effective in detecting *Plasmodium* in suspected malaria patients than microscopy. It detected 13.1% (39/297) positives which had been confirmed negative by Giemsa microscopy. This high rate of misidentification by microscopy was associated with the difference in detection limit of the two methods, and skills and experiences of microscopists to detect and identify *Plasmodium* parasites [16]. Giemsa microscopy could not detect parasite densities below 100 parasites/μl of blood while nested PCR had the ability to detect *Plasmodium* parasitaemia as few as one parasite/μl of blood [11,19]. The LAMP method had a similar detection threshold with nested PCR [22]. In this study, malaria NINA-LAMP demonstrated diagnostic sensitivity similar to that of nested PCR and superior to that of microscopy. Therefore, the NINA-LAMP method could be a practical alternative to PCR method to avoid misidentification of malaria by microscopy in endemic areas and detection of submicroscopic infections in low transmission settings.

The method relied on isothermal amplification in a NINA heater, which permits POCT in low resource settings. The NINA enabled electricity free reaction conditions comparable to a thermocycler or water bath. The NINA-LAMP test procedure used in this study did not require special equipment and can be used in remote malaria endemic areas. The test procedure was also done with short-term training and no previous experience. Negative and positive controls were used for interpretation of sample test results. For running one batch of five tests, the whole test procedure took 60–80 minutes. Considering the advantages of rapid amplification, simple operation and easy detection, NINA-LAMP has potential applications for clinical diagnosis and surveillance of malaria in developing countries including Ethiopia without requiring electricity, sophisticated equipment or skilled personnel. Limitations of this study include the use of microscopy for case detection and the possible bias that may introduce, and also the failure to obtain “asymptomatic” or low parasitemia cases. Furthermore, sample preparation still requires a centrifugation step.

The NINA-LAMP method was performed in three separate steps namely DNA extraction, addition to the LAMP reaction tube, followed by NINA-based amplification. Combining the three steps may reduce cost, turnaround time, and possible risk of contamination. This study relied on commercially available LAMP kits from Eiken Chemicals, Japan and a NINA instrument from PATH, USA. Modification of this NINA prototype to include direct sample addition could simplify the procedure and eliminate contamination risk for the diagnosis of malaria in remote low-resource settings.

In conclusion, NINA-LAMP performed well in comparison to nested PCR for the diagnosis of malaria suspected febrile patients in Ethiopia. NINA-LAMP was highly sensitive for detection of *Plasmodium* parasites. The method was simple and experience was not required to perform the test procedure. The result of the test was also easy to read and interpret as compared to routine diagnostic method by Giemsa microscopy. The lyophilized reagents were thermostable. Future studies will aim to evaluate the potential for NINA-LAMP to detect asymptomatic or low parasitemia cases missed by microscopy, a central focus of the National Control Programme in the next decade.

## Abbreviations

DNA: Deoxyribonucleic acid
EDTA: Ethylene diamine tetraacetic acid
FMOH: Federal Ministry of Health
LAMP: Loop-mediated isothermal amplification
NINA: Non-instrumented nucleic acid amplification
n-PCR: Nested-polymerase chain reaction
NPV: Negative predictive value
PATH: Program for appropriate technology in health
POC: Point of care
PPV: Positive predictive value
RDT: Rapid diagnostic test
RNA: Ribonucleic acid
SPSS: Statistical package for social sciences
WHO: World Health Organization
